# A study of implementation of national language policies in a local higher institution: a case study from China

**DOI:** 10.1186/s40064-016-3731-y

**Published:** 2016-12-01

**Authors:** Xiaorui Chen

**Affiliations:** 1 School of International Studies, Binzhou Medical University, Yantai, Shandong People’s Republic of China; 2 Department of English Language and Literature, Gachon University, Seongnam-si, Gyeonggi-do Korea

**Keywords:** College English Curriculum Requirement, Education reform, Course setting, Binzhou Medical University, College English

## Abstract

**Background:**

College English syllabi have been a guide for English teaching in China for decades. In order to explore how the 2007 version of the College English Curriculum Requirement can succeed in local implementation, this case study chose Binzhou Medical University as a participant institution to find out the influential factors of the implementation of national language policies.

**Case presentation:**

Binzhou Medical University implemented the College English Curriculum Requirements of 2004 and 2007 versions gradually in terms of course setting. Differentiating Instruction was conducted in 2005 and improved in 2012. It was warmly welcomed by students. The diversity of English courses was advocated in 2007, and the management of the courses was enhanced in 2012. The practices tended to satisfy students’ continuous change of academic interests. English for Specific Purpose instruction was introduced in 2012, but further improvements were required. Internet-based teaching system was introduced in 2014 and micro-lessons were strongly advocated in 2015. The effects remained to be seen in the future.

**Conclusions:**

Several factors influenced the College English reform. First, the ideology of national and local administrations was a key factor for the College English reform. Second, professional identity of the teachers affected the development of the course setting. Third, globalization attracted attention to students’ communication competence and provided new ways for training teachers. The findings of this article can be valuable references for drawing up an updated syllabus.

## Background

College English has been a compulsory course for non-English major students in China for decades. According to Chu (2011), the development of College English Syllabi has gone through five stages: Budding Stage, Starting Stage, Developing Stage, Exploring Stage, and Mature Stage.

### The budding stage (from the building of new China to the late 1970s)

Though Russian was the dominant foreign language taught in the early years of new China for historical reasons, some higher institutions started College English as a public course from 1956. Then, it gradually gained its popularity after 1960 (Chu 2011). In 1962, the Chinese Ministry of Education (CME) issued the first English Teaching Syllabus to give a unified direction for the nationwide English education of 5-year-universities of technology. The sole objective of the first syllabus was “to make students well-prepared for reading major-related books and magazines in the future” (FLTRGSJTU 1963). In 1966, college English education turned from its budding state to a paralyzed state owing to the Cultural Revolution. Then, the first English Teaching Syllabus was totally denied by the government (Li 2008), and this situation continued until 1978 when the Revolution ended.

### The starting stage (from 1978 to the mid-1980s)

Since China opened its door to the rest of the world in 1978, the importance of English has become a key factor to the country’s development and to its economic and social mobility (Jeon and Lee 2006). This ideology of national administration towards English led to the revival of College English in universities. In 1980, Public English Teaching Syllabus (Science and Engineering) was published by People’s Education Press, and it was greatly similar to the Syllabus of 1962. However, it lasted for only 5 years due to some defects of teaching requirements and evaluation methods (Li 2008).

### The developing stage (from the mid-1980s to the mid-1990s)

College English Teaching Syllabus (CETS) of the 1986 version was generated with the government’s firm determination to provide a reformation of comprehensive English teaching and learning. The purpose of it was to get rid of the out-of-date Syllabus, backward teaching method, and crude teaching equipment (RTGCLASETS 1986). The CETS of 1986 was regarded as the greatest Syllabus compared with the previous ones, which were lacking in clarity in teaching objectives, contents and methods (Li 2008). During this period, though reading had its dominant place, the importance of listening was gradually highlighted (Wei 2009).

### The exploring stage (from the mid-1990s to the early 2000s)

Although the CETS of 1986 was satisfactory, the results of its implementation and its weaknesses needed to be examined after a period of 10 years and before the forthcoming of the new century. After many seminars and conferences on College English education, a new syllabus was proposed in 1999 as a leading program to guide College English education (Chu 2011). Reading skills were still primary in the 1999 version, and listening and speaking skills were regarded as secondary (RTGCETS 1999). Significantly, it was the first time to include speaking skills in English teaching objective.

### The mature stage (from the mid-2000s to 2015)

With the development of technology and globalization, CME published a pilot of College English Curriculum Requirement (CECR) in 2004, as a replacement for the CETS of 1999, to satisfy the demands for talent cultivation in a new era of China (Chu 2011). The CECR (2004) especially emphasized the importance of listening and speaking skills, and explicitly proposed that one of the teaching objectives was for the successful oral communication in English, which was and is still relevant for social needs (HEDEM 2004). After three and half years, CME published another revised CECR as a formal version of CECR in September, 2007. The CECR (2007) lasted for 8 years, and still influences College English education in the present time.

Thus, the historical development of College English discussed above showed that syllabus for English education was formulated according to the social environment and the ideology of the administrators at the time, on the matter of guiding College English teaching. Moreover, the changes of teaching syllabi usually gave rise to teaching reforms.

The fact that the CECR (2007) has influenced College English teaching so far demonstrates that it has successfully played the role of guidance. This article conducted a case study to show how the CECRs (2004, 2007) directed College English teaching at Binzhou Medical University (BMU), a non-key university in the east of China, and how the change of syllabi from the CECR (pilot, 2004) to the CECR (2007) led to teaching and learning modifications at BMU. The research might be true of other universities since non-key universities are in the majority in Chinese higher institutions (Cheng 2014).

## Methods

### Participants

 Albert was in his forties. He was a dean of Teaching Affairs at BMU, and worked in this department for nearly 10 years. Therefore, he witnessed the College English reforms at BMU and participated in some decision-making processes of the reforms. Alice was in her late thirties. She was an administrator of elective courses, and worked in Teaching Affairs at BMU for 5 years. Bella was in her fifties. She had been a leader of College English office at BMU for 14 years, and taught at BMU for more than 20 years. She was a witness of many College English reforms in national and local levels. At the same time, she was a key member to lead College English teaching reforms at BMU. Care, Carol, Catherine, Cathy, Charlotte and Crystal were all in their thirties. They taught College English at BMU from 8 to 12 years. All of them obtained Master Degrees in English literature or linguistics, while no one had a dual degree. All of them got married and had little kids. In addition, all the participants’ names are pseudonyms for ethical consideration.

### Data collection and analysis

Data were collected from text-based artifacts and four semi-structured interviews. Text-based artifacts refer to already existing documents, archives, or any text-based materials produced in research process (Yang et al. 2013). They are commonly used as sources of evidence in qualitative data collection concerning policy studies and text studies (Duff 2014). In this research, text-based artifacts included the documents of the CECR of 2004 and 2007, the English syllabi and course notices of BMU, and the archives of work plans and work reports by the dean of Teaching Affairs and the leader of College English office at BMU. These materials were closely related to research questions. Therefore, they could reflect the process of College English reforms at BMU. The collection of text-based artifacts started from June 2015, and was finished by the end of the year. The first interview was conducted with the dean of Teaching Affairs in October 2015. Main questions addressed in the interview included: what has BMU done for the changes in College English course setting in the past 10 years? How are these reformed courses administered? Are they effective or not? If not, what are the problems? According to the dean’s suggestion, the second interview was conducted with the administrator of elective courses the following day. Main questions were concerned with what the regulations of elective courses setting were, how elective courses were administered, and whether they were satisfactory. The third interview was conducted with the leader of College English office. Questions were mainly about how EMP instruction was going, whether there were any obstacles, and what kind of development of internet-based courses had gone through. The fourth interview with College English teachers was a group interview. It was focused mainly on the perceptions of the ESP instruction. The last two interviews were both conducted in November 2015.

The course setting parts of the CECR of 2004 (pilot) (HEDEM 2004: 8–9) and the CECR of 2007 (HEDEM 2007: 16–18) were manually coded and compared to elicit themes related to course setting reforms. English syllabi and course notices of BMU were categorized according to the themes elicited above. The purpose was to find out the local implementation of national policies. Archives of the work plans and work reports by the dean of Teaching Affairs and the leader of College English office at BMU were manually coded according to the built-up themes. The purpose was to search for evidences which showed the process and problems of the CECRs implementation. All semi-structured interviews—each lasted about one and a half hours—were audio-recorded and transcribed. All participants spoke in Chinese. Through careful reading, all the transcriptions were manually coded in chunks in order to get a clear idea of the perceptions of the CECRs implementation. At last, relevant transcriptions were translated in English.

## Case presentation

Both CECR (pilot, 2004) and CECR (2007) consisted of six parts: the nature and the target of College English teaching; teaching and learning requirements; course setting; teaching mode; teaching and learning evaluation; and teaching management. Due to word limits, the article only focused on course setting. Course setting, an explicit aspect of English course, can reflect social needs and the ideology of administrators. College English teaching at BMU was included as a case to illustrate the local implementation of the national language policy.

### A comparison between CECR (pilot, 2004) and CECR (2007)

#### The similarities of course settings

CECR (pilot, 2004) and CECR (2007) both emphasized that course settings should satisfy the characteristics of different students and the demands of students with different English levels. In addition, course setting should meet the needs of students with different majors, which was a new point mentioned for the first time by CME. Before CECR (pilot, 2004), the main College English education was English for General Purposes (EGP), while CECR (pilot, 2004) could be regarded as the starting point to propose the necessity of English for Specific Purposes (ESP) (HEDEM 2004, 2007). The need for international cooperation and globalization, after joining World Trade Organization (WTO) in 2001, was one of the reasons why ESP was proposed. Since then, the Chinese government has increasingly realized a lack of human resources with specific English knowledge, such as Business English and Legal English (Cai 2002). This ideology led to the proposal of involving ESP in College English teaching in CECR (pilot, 2004). With the recognition of the importance of ESP (Sun and Li 2011; Wang 2010), CECR (2007) kept on emphasizing ESP teaching.

#### The differences of course settings

Apart from the traditional course settings of listening, speaking, reading and writing, CECR (pilot, 2004) put emphasis on the computer-based and internet-based courses, but it suggested that teachers’ face-to-face tutorials was necessary for this kind of courses. In addition, it mentioned that teachers should get paid for the face-to-face tutorials, and that students should take examinations in the computer-based and internet-based courses, with its scores taken into credits. CECR (pilot, 2004) also suggested that the traditional class (teacher-students) should be kept for reading, writing and translation teaching, and outstanding or famous teachers should be employed for these courses (HEDEM 2004). An assumption could be made from the last suggestion: though CECR (pilot, 2004) brought in the concept of computer-based and internet-based courses, it assumed these new forms were more appropriate for teaching listening and speaking, while reading, writing and translation should be taught in traditional classrooms. Moreover, teachers still functioned in some extent (as assistants) in computer-based and internet-based courses. After joining WTO in 2001, China obtained new opportunities for worldwide cooperation, which promoted greater development of technology and accelerated globalization. At that time, there were already several English learning software systems and on-line English textbooks in foreign countries (Li 2008). After several famous Chinese Press companies organized national experts, and experts aboard conducted research on the resources, China decided to take action in developing computer-based and internet-based courses by itself (Li 2008). However, this kind of courses was implemented gradually. CME chose 180 institutions of higher education as pilot schools to see its effects (Li 2008). Of course, teachers’ role could not be completely denied in the pilot phase. Therefore, CECR (pilot, 2004) still valued teachers’ role in the computer-based and internet-based courses, saying that face-to-face tutorials from teachers were necessary in such courses.

CECR (2007) was a modified version of CECR (pilot, 2004). Some revisions were made in terms of course setting. CECR (2007) still put emphasis on computer-based and internet-based courses, but it didn’t require face-to-face tutorials from teachers for this kind of courses as well as examinations of the computer-based and internet-based courses. The revision was due to the ideas of the research findings from pilot schools (Li 2008). It also paved the way for autonomic learning in China. A great change of CECR (2007) was that it no longer confined computer-based and internet-based courses to listening and speaking (HEDEM 2007). As long as advanced technology could be used in College English teaching, there was no need to demand on how to use it. In this case, the increasingly diverse technology could be applied not only to listening and speaking, but also to reading and writing. The change indicated that administrators in CME had more mature cognition towards the application of technology to English teaching than before, since they regarded technology no longer as an aid but a main teaching and learning tool (Chen and Gu 2008).

To sum up, the trend of College English reform in China is to combine traditional courses with computer-based and internet-based courses, and to satisfy the demands of the students with different English levels, interests and majors in order to keep up the pace with the development of society and technology. Hence, there were four general themes elicited by comparison: technology-based courses, ability-based courses, interest-orientated courses, and major-based courses. However, in what ways should we meet the requirements by following the current trend? There was no explicit instruction on the concrete practices in the two CECRs. Higher institutions had to decide their own ways according to their practical conditions. BMU has gradually met the requirements by making use of differentiating instruction, diverse elective courses, English for Medical Purposes (EMP) instruction and the introduction of new learning software and micro-lessons.

### Implementation of national language policy: a case study of BMU

CECR (pilot, 2004) and CECR (2007) played the role of guidance during their times. Therefore, universities exerted their efforts to follow. However, in reality, many universities could not fulfill or spend a long time fulfilling the requirements set by the CME. In this sense, it was worth studying the question “what is the process for universities to catch up with the requirements set by the CME?” to find out the influential factors of the implementation of national language policy.

According to CECR (pilot, 2004) and CECR (2007), universities should set their own requirements according to the national language policy suitable for their own conditions. However, BMU only added the names of textbooks and the titles of each unit to CECR (pilot, 2004) and CECR (2007) to form its own “syllabi” (unpublished files). In this case, CECR (pilot, 2004) and CECR (2007) were not localized by BMU, but were applied directly. This practice demonstrated the consistency of national and local ideology toward College English. Therefore, BMU has long been exerting its efforts to meet the requirements. Corresponding to the discussion above, the following contents only focused on course setting at BMU from 2004 to 2015.

#### Differentiating instruction

Since CECR (pilot, 2004) put an emphasis on satisfying the demands of students with different English levels (HEDEM 2004), BMU began to implement Differentiating Instruction from freshmen students of 2005 according to the 2005 work report of the dean of Teaching Affairs at BMU. In the interview, the dean described how they conducted the Differentiating Instruction. He said, “All freshmen students took an English examination after being admitted to BMU, and roughly 240 students (totally 3000 students) with higher scores were placed in A-level classes which consisted of 6 classes, 40 students for each class, while the other students were placed in B-level classes.” For the course arrangements for Differentiating Instruction, the leader of College English office depicted, “the textbooks are the same for A-level and B-level classes. However, A-level classes only learn one passage in each unit of the intensive textbook, well, there are two passages in total, while B-level classes learn two passages in each unit. Therefore, A-level classes will touch upon more advanced materials than B-level classes within 2 years of College English courses.”

The implementation of Differentiating Instruction enabled the progress of students with higher level of English proficiency, and at the same time, it also provided students with lower level of English proficiency with appropriate instruction. Then a further reform was made in 2012. From then on, Differentiating Instruction became dynamic at BMU. “Dynamic” here refers to the mobility of students between A-level and B-level classes according to the 2012 work report of the dean. In the interview, he further explained, “if a student in an A-level class cannot follow the class schedule, he or she is allowed to choose B-level class to continue their English studies, while if a student in a B-level class feels it is a waste of time to study in his/her class because of the slow schedule, he or she is allowed to go to A-level class”. This flexible administration took full consideration of students’ English levels. Therefore, it was warmly welcomed by the students, according to the dean.

#### Diversity of English courses

Although CECR (pilot, 2004) put emphasis on the diversity of English courses (HEDEM 2004), BMU turned its attention to diversity until 2007. Because CECR (2007) still focused on the diversity of English courses (HEDEM 2007), according to the dean of Teaching Affairs, the administrators of BMU advocated setting English elective courses with no regard to course contents in order to keep up with the pace with the trend of College English reform. However, there were some problems with English elective courses. An interview with one administrator of elective courses revealed that some teachers were not fully qualified for their courses, and they chose to set their courses solely out of their individual interest. The most striking problem, the interviewee emphasized, was that nearly all the elective courses lacked continuity. Therefore, students couldn’t benefit further from the courses. In 2012, in order to encourage the normalization and continuity, BMU provided funds for each elective course, demanding the teachers to build e-classes for their courses and encouraging the teachers to renew learning materials continuously (TAOBMU 2012). Furthermore, there was a regulation for elective courses: if an elective course is chosen by less than 20 students, the course will be canceled (TAOBMU 2015a), which undoubtedly put students’ interests and needs in the first place. Now most of the elective courses have the characteristics of diversity, normalization and continuity. The administrator of the elective courses said, “now BMU still enrolls new courses every 3 year and at the same time cancels unwelcomed courses.” This dynamic administration of courses tended to satisfy students’ continuously changing academic interests.

#### ESP instruction

BMU began its course of ESP in 2012 according to 2012 work report of the dean in Teaching Affairs. However, unlike the setting of Differentiating Instruction and English elective courses, ESP instruction was initially proposed by the leader of College English office, not the administrators at BMU. Several College English teachers in BMU shared their common concerns in the interview. They said, “since the trend of ESP instruction was wildly spread in China, several medical major teachers at BMU, who had learning or working experiences in foreign countries, began to teach their major courses in English in 2011. We suddenly felt we were at risk of losing our jobs, or at least, our status at BMU was severely threatened. Our leader also perceived the problem. Therefore, she suggested, to the dean of Teaching Affairs, that EMP course should be set by College English teachers.”

The objective of EMP was to give students general introduction of medical knowledge through English materials as a transition to major courses in English by major teachers (Chen 2013). In this situation, English teachers were forced to participate in Medical English Reading instruction without any professional training in advance (Li 2012). The 2014 work plan of the leader of College English office stated that in order to solve the problem of the qualifications of EMP teachers, EMP teachers should choose topic of their own interest in one field of medicine, such as pathology, physiology, anatomy, etc., to engage in major teachers’ activity of lesson preparations for the knowledge of medicine and for a clear idea of what should be taken into consideration as a transitional knowledge. However, most of the EMP teachers in the interview complained that “while the idea of cooperation between major teachers and EMP teachers is good, we really have little time to devote ourselves to the cooperation, let alone a long time of persistence. Imagine regular teaching, other office work, meetings, family, kids and a brand new major!” As a consequence, EMP instruction still focused on meta-linguistic level, such as vocabulary, grammar and text translation according to the 2015 work report of the leader in College English office. The other reason for the slow improvement of EMP instruction was that there was lack of need analysis on EMP (Gao 2012). EMP is different from other ESP courses, such as Business English and Legal English, which can be promoted greatly by globalization. EMP was not in urgent need at BMU. According to EMP/College English teachers, students had little chance to have contact with patients from foreign countries, or to work in foreign countries in the future.

Furthermore, BMU had limited contact with western universities, and there was no exchange program with English speaking countries for students of medical majors. Therefore, the leader in College English office said, “EMP instruction today stays almost in nearly the same condition as it was 3 years ago since it was rarely needed by both teachers and students.” In the interview, EMP teachers said that they were uncertain if the contents they had been teaching were befitting, while they believed that students’ low attendance rate showed students’ doubt about the benefits of the EMP course for their future career.

Although there was lots of need to improve the EMP course at BMU, this reform was made due to the consistency of national and local ideology toward ESP. Both national administrators and College English teachers thought the setting of EMP course to be necessary, even though they had different reasons: one for human resources, and the other for job security.

#### Introduction of new learning software and micro-lessons

The last point of course setting reform is related to technology. Firstly, the improvement of technology, which is relevant to College English instruction at BMU, is described here. College English is a public discipline at BMU. Although it is not a major course, and BMU tended to spend relatively less budget on it compared with that of medical courses (unpublished files), the hardware of College English course had always been under construction. According to 2015 work report by the dean of Teaching Affairs, there were three language labs in 2004, five in 2005, seven in 2006, and nine in 2009 until now (roughly 6000 students use language labs each year). In an interview, the leader of College English office made a description of how language labs had been operated. She said, “language labs at BMU had been used for listening and speaking classes, and after the classes were over they were locked. Students could not get access to Internet in Language labs until 2013. However, it seemed that both teachers and students didn’t need access to the Internet as teachers used disks (CDs and flash drives) companied with listening textbook for the whole class. So far, there is no computer-assistant and internet-assistant equipment supports autonomic learning.”

Anyway, there was a change in 2014. According to the leader of College English office, BMU approved of purchasing a writing correction system “Smartpigai”. What teachers should do was to give a topic for writing, and then writing code was created automatically. What students needed to do was to just type in the code and start to write. After submitting their writing, they would get scores and detailed comments for their writing. Students could correct their writing according to the suggestions offered by the system, and submit again to see if they could get higher scores, and there was no limit for the number of revisions. Therefore, education reform could not achieve obvious improvements without assigning too much budget for it.

In 2012–2013, micro-lessons began spreading in Chinese universities (Zhang and Qian 2013). In order to keep up with the trend, BMU waged a campaign to train both English teachers and major teachers for a blended teaching mode since October 2015 (TAOBMU 2015b). The dean of Teaching Affairs stated in an interview that BMU supported micro-lessons by purchasing equipment for videotapes or paying for videotapes made by other companies. All the micro-lessons were assumed to put on the educational platform of BMU as materials for students’ autonomic learning. Thus, the process of reform mentioned above revealed that the ideology of local university towards internet-based courses determined whether the national policy could be implemented in time, or implemented later.

## Conclusions

From the case study of BMU, some implications can be made concerning national policy implementation and College English reform.

Firstly, the ideology of administration is a key factor for College English reform. There are two layers of administrations: the national administration of the country and the local administration of a university. Both of the ideologies should be consistent, and only one party’s effort on certain reform cannot lead to its success due to the lack of support, or the lack of budget, or for both. Lack of support in turn affects renewal of old equipment, introduction of advanced technology, and teacher training.

Secondly, teachers’ professional identity affects the development of course setting, such as the EMP course which was proposed by College English teachers to keep their social status in their workplace. However, a course may be instructed in a dissatisfying way due to teachers’ lack of academic theories of a new trend. Therefore, teachers’ training and self-study in new academic theories are important to put prevalent theory into practice. Cooperation between College English teachers and major teachers are assumed to be useful to cultivate students’ academic English competence, but it needs systematic evaluation to confirm that it meets ESP teachers’ demands (Butler 2004). Currently teachers’ limited devotion to the cooperation and students’ shortsighted view of their future need for ESP are barriers for the ESP instruction. Therefore, need analysis should be conducted on both with teachers and students. Meanwhile, English teachers should be divided into EGP and ESP teachers to lessen their burden of devoting themselves to both EGP and ESP.

Lastly, globalization plays a positive role in College English reform. It makes administrators and teachers pay more attention to cultivating students’ communication competence, which is mentioned both in CECR (pilot, 2004) and CECR (2007). In addition, globalization provides a new way for teacher training, such as Exchange Programs by Bureau of Educational and Cultural Affairs (Exchange programs 2016), and self internet-based learning, such as Massive Open Online Courses (MOOC).

This paper gives an overview of the similarities and differences of CECR (pilot, 2004) and CECR (2007) to demonstrate the current trend of College English instruction. A case study from BMU demonstrates clearly how a non-key university in China exerts its effort to catch up with the requirements set by the country. The ideology of administration, teachers’ social identity and globalization are all factors that influence the course setting of College English. Therefore, making full use of positive parts of these factors may bring a bright future for College English instruction and reform, and be helpful for achieving two layers of significance of English education: for individual development and for social development (Lin 2011).

However, this study is only conducted from the perspective of course setting in CECRs. Therefore, it lacks a holistic view of investigating the trend of current College English teaching. Also, the study is concerned with a case of non-key university, which cannot represent the process of College English reform in other types of higher institutions. Hence, further study can focus on other parts of the CECR. The findings may be a helpful supplement for those relevant to this article. Case studies from other types of colleges and universities are also of necessity to find out the problems and barriers confronted by them, because it will be beneficial to find out solutions through inter-institutions communication. At the same time, findings of these case studies can be regarded as valuable materials to provide references for drawing up an updated CECR in the future.

## Abbreviations

BMU: Binzhou Medical University
CECR: College English Curriculum Requirement
CETS: College English Teaching Syllabus
CME: Chinese Ministry of Education
EGP: English for General Purposes
EMP: English for Medical Purposes
ESP: English for Specific Purposes
MOOC: Massive Open Online Courses
WTO: World Trade Organization

## References

[CR1] Butler YG (2004). What level of English proficiency do elementary school teachers need to attain to teach EFL? Case studies from Korea, Taiwan, and Japan. TESOL Q.

[CR2] Cai JG (2002). Pressure on the current college english teaching. Foreign Lang Teach Res.

[CR3] Chen XR (2013). A study of the effect on the application of CBI teaching mode of college English in medical universities. Chin J Med Educ Res.

[CR4] Chen JL, Gu ZZ (2008). More reasonable and precise requirements with realizing directions: interpreting the 2007 College English Curriculum Requirements. Comput Assist Foreign Lang Educ China.

[CR5] Cheng Y (2014). A study on the historical changes of key university policy in China during the Past 30 Years. J Zhaoqing Univ.

[CR6] Chu RL (2011). A review of College English teaching in the past 50 years. Shaanxi Educ (higher education version).

[CR7] Duff PA (2014). Case study research in applied linguistics.

[CR8] Exchange Programs (2016) https://exchanges.state.gov/non-us. Accessed 20 August 2016

[CR9] Foreign Language Teaching and Research Group of Shanghai Jiaotong University (1963). English Teaching Syllabus (pilot): 240 credit hours.

[CR10] Gao JJ (2012). A review of research into needs analysis in ESP. Chin J ESP.

[CR11] Higher Education Department of Education Ministry (2004). College English Curriculum Requirements (pilot). Chin Univ Teach.

[CR12] Higher Education Department of Education Ministry (2007). College English Curriculum Requirements.

[CR13] Jeon M, Lee J (2006). Hiring native-speaking English teachers in East Asian countries. Engl Today.

[CR14] Li J (2008) College English teaching and learning research of the People’s Republic of China. Dissertation, East China Normal University

[CR15] Li L (2012). Obstacles for General English teachers to take up English for Medical Purposes teaching and the corresponding strategies. Chin J ESP.

[CR16] Lin P (2011). English language ideologies in the Chinese foreign language education policies: a world-system perspective. Lang Policy.

[CR17] Revision Task Group of College English Teaching Syllabus (1999). College English Teaching Syllabus.

[CR18] Revision Task Group of College Liberal Arts and Science English Teaching Syllabus (1986). College English Teaching Syllabus (Liberal Arts and Science).

[CR19] Sun YZ, Li LW (2011). The direction of Chinese English majors and College English teaching reform with CBI and ESP. Foreign Lang Res.

[CR20] Teaching Affairs Office of Binzhou Medical University (2012) Notice of the 2012 elective course construction project. http://jwc.bzmc.edu.cn/info/1013/1024.htm. Accessed 19 Jun 2012

[CR21] Teaching Affairs Office of Binzhou Medical University (2015a) Notice of starting elective courses. http://jwc.bzmc.edu.cn/info/1013/1723. Accessed 9 Sep 2015

[CR22] Teaching Affairs Office of Binzhou Medical University (2015b) Blended courses design and training of network teaching platform application. http://jwc.bzmc.edu.cn/info/1012/1748.htm. Accessed 27 Oct 2015

[CR23] Wang SR (2010). Develop ESP teaching and promote ESP research in China. Chin J ESP.

[CR24] Yang LX, Wang SE, Chang HC, Sheng J (2013). Qualitative research in applied linguistics.

[CR25] Zhang YC, Qian YY (2013). The resource construction of microlecture at home and abroad and its latest application development. China Acad J Electron Publ House.

